# Parkinson’s Disease Etiology: Insights and Associations with Phosphate Toxicity

**DOI:** 10.3390/ijms23158060

**Published:** 2022-07-22

**Authors:** Ronald B. Brown

**Affiliations:** School of Public Health Sciences, University of Waterloo, Waterloo, ON N2L 3G1, Canada; r26brown@uwaterloo.ca

**Keywords:** Parkinson’s disease, phosphate toxicity, mitochondrial dysfunction, calcium phosphate, tauopathy, ectopic calcification, bone mineral disorders, vitamin D, sarcopenia, cancer

## Abstract

The present paper investigated the association of Parkinson’s disease etiology with phosphate toxicity, a pathophysiological condition in which dysregulated phosphate metabolism causes excessive inorganic phosphate sequestration in body tissue that damages organ systems. Excessive phosphate is proposed to reduce Complex I function of the mitochondrial electron transport chain in Parkinson’s disease and is linked to opening of the mitochondrial permeability transition pore, resulting in increased reactive oxygen species, inflammation, DNA damage, mitochondrial membrane depolarization, and ATP depletion causing cell death. Parkinson’s disease is associated with α-synuclein and Lewy body dementia, a secondary tauopathy related to hyperphosphorylation of tau protein, and tauopathy is among several pathophysiological pathways shared between Parkinson’s disease and diabetes. Excessive phosphate is also associated with ectopic calcification, bone mineral disorders, and low levels of serum vitamin D in patients with Parkinson’s disease. Sarcopenia and cancer in Parkinson’s disease patients are also associated with phosphate toxicity. Additionally, Parkinson’s disease benefits are related to low dietary phosphate intake. More studies are needed to investigate the potential mediating role of phosphate toxicity in the etiology of Parkinson’s disease.

## 1. Introduction

Parkinson’s disease (PD) is the second most prevalent neurodegenerative disorder, after Alzheimer’s disease, and hallmarks of PD include neuronal losses in the substantia nigra causing deficiencies in dopamine [1]. In addition to movement disorders (rigidity, resting tremor, and bradykinesia), conditions associated with PD include cognitive impairment, sleep disorders, and depression [2]. Globally, the burden of PD has more than doubled over the past generation and is predicted to increase substantially [3]. No curative treatments exist for PD, and “modifying disease progression and further delaying disability are the key unmet needs to be addressed by current and future research efforts” [1]. Ongoing clinical trials are testing whether dopamine production can be strengthened through central nervous system gene therapy that transfers glial-cell-line-derived neurotrophic factor (GDNF) to PD patients [4]. Although results of the gene therapy indicate clinical benefits and improved response to levodopa medication, “it remains to be seen whether the many debilitating non-motor features will also show relevant responses to treatment”.

The present paper investigated the association of PD etiology with phosphate toxicity, a pathophysiological condition resulting from excessive inorganic phosphate (Pi) sequestered in body tissue [5]. Phosphorus in the form of phosphate (PO_4_) is an important micronutrient that serves many functions in the body, and Pi homeostasis is normally regulated by a sensitive network of hormones released by the kidney, bone, parathyroid gland, and intestines. Under conditions of burdened renal function, Pi from dietary sources may accumulate in extracellular and intracellular tissue, causing toxic effects on cells of the body’s major organ systems. Evidence reviewed in the present article supports a pathophysiological mechanism in which phosphate toxicity from dysregulated phosphate metabolism potentially contributes to the etiology and progression of PD. Using a grounded theory approach to rigorously and objectively review relevant concepts from the research literature [6], an iterative process of comparative analysis induced a theory grounded in evidence, offering novel insights and proposed associations linking PD with phosphate toxicity.

## 2. Calcium Phosphate and Mitochondrial Dysfunction in PD

Experiments demonstrated that brain and neuron mitochondria are capable of forming “remarkably high levels” of calcium phosphate precipitates within the mitochondrial matrix, which are “retained in damaged mitochondria for prolonged periods” [7]. Mitochondrial precipitates are “composed primarily of tribasic calcium phosphate [Ca_3_(PO_4_)_2_] and/or dibasic calcium phosphate (CaHPO_4_)”. Of relevance, mitochondrial damage involving accumulated Pi was found in cultured pancreatic beta cells and islet cells [8,9], which is associated with reduced insulin biosynthesis and secretion, reactive oxygen species, and cell apoptosis in diabetes mellitus. This pathophysiological mechanism appears strikingly similar to reduced dopamine biosynthesis and secretion in the substantia nigra associated with PD, suggesting that increased Pi concentrations and calcium phosphate accumulation in the mitochondrial matrix may be included among shared etiological factors in PD and DM [10].

A recent systematic review and meta-analysis confirmed a significant 21% increased odds of PD associated with type II diabetes mellitus (T2DM) [11]. Experiments conducted on the mitochondrial electron transport chain in guinea pig hearts found that increased accumulations of calcium phosphate inhibited proton pumping at Complex I (NADH CoQ reductase) and reduced ATP synthesis necessary to power cellular functions [12]. Researchers suggested that calcium phosphate particles act as a physical barrier within the cristae of the mitochondrial inner membrane, which disrupts the operation of Complex I. Coincidently, a postmortem study found “a specific defect of Complex I activity in the substantia nigra of deceased patients with Parkinson’s disease” [13]. Additionally, the researchers noted that an inhibiting effect on Complex I from the neurotoxin derived from 1-methyl-4-phenyl-1,2,3,6-tetrahydropyridine (MPTP) was found to cause symptoms of PD, which “adds further support to the proposition that Parkinson’s disease may be due to an environmental toxin with action(s) similar to those of MPTP”.

Reduction in Complex I function in PD is linked to opening of the mitochondrial permeability transition pore (mPTP), allowing an influx of ions and small molecules, which is associated with increased reactive oxygen species (ROS), inflammation, DNA damage, mitochondrial membrane depolarization, and ATP depletion causing cell death [14,15,16]. “Phosphate activation of the mitochondrial permeability transition pore opening is well documented” [17]. Of relevance, reduction of polyphosphate (polyP)—a phosphate chain linked by ATP bonds in mitochondria—reduces calcium-induced mitochondrial permeability transition [18]. Furthermore, researchers using fluorescent probes estimated higher levels of endogenous polyP in models of living cells “with Parkinson’s disease related mutations” [19]. Studies are needed to further investigate the role of high Pi concentration and accumulated calcium phosphate precipitates on mitochondrial Complex I function and the opening of the mPTP in patients with PD.

**Figure 1:**
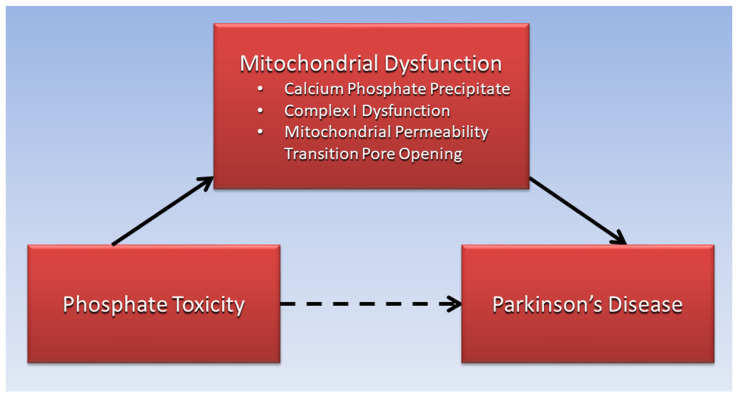
Mitochondrial dysfunction is proposed to mediate the association of phosphate toxicity with Parkinson’s disease.

The present paper’s proposed association of phosphate toxicity with PD, mediated by mitochondrial dysfunction, is illustrated in Figure 1. The remaining sections of this paper review comorbid conditions in patients with PD, which involve systemic dysregulation of phosphate metabolism and phosphate toxicity associated with PD etiology.

## 3. Tauopathy, Diabetes, and PD

PD is associated with α-synuclein and Lewy body dementia, with tau aggregation forming neurofibrillary tangles—a secondary tauopathy potentially caused by pathological exposures [20,21]. Of relevance, tauopathies are associated with tau protein hyperphosphorylation, whereby phosphorylation at multiple sites leads to altered protein function that “may be involved in the pathogenesis of neurodegenerative disorders” [22]. In vitro experiments confirm that α-synuclein phosphorylation at serine 129 induces pathogenic changes found in Lewy body formation deposited within PD patient brains, but how this phosphorylation occurs in vivo “remains a mystery”, which researchers speculated is related to “phosphokinase upregulation”, the enzyme responsible for phosphorylation [23]. Research is needed to investigate tauopathy hyperphosphorylation and synucleinopathies potentially associated with in vivo exposure to large amounts of dysregulated inorganic phosphate and upregulated phosphokinase levels. Hypothetically, excessive Pi may be a rate-limiting factor in tauopathies, similar to Pi’s potential rate-limiting role in tumor growth [24] (see Section 7). That is, tauopathies may be reduced if phosphate metabolism is well regulated and an excessive supply of Pi is less available for hyperphosphorylation.

Moreover, tauopathy is associated with inflammation, oxidative stress, impaired insulin signaling, and insulin secretion in diabetes [25], and greater amounts of total tau and phosphorylated tau were found in cerebrospinal fluid of patients with type 2 diabetes (T2DM), which researchers suggested may be related to neurodegeneration [26]. Pre-existing T2DM is also associated with faster progression and reduced survival in PD, contributing additional support for a pathogenic pathway shared by diabetes and PD [27], with potential mediation by dysregulated Pi metabolism. Additionally, a retrospective cohort study in England found an increased risk of subsequent PD in 2,017,115 individuals with T2DM, which the researchers said may reflect “disruptive shared pathogenic pathways” [28]. A study of 451,743 individuals from the European Prospective Investigation into Cancer and Nutrition (EPIC) found an association of increased soft drink consumption, often high in phosphoric acid, with increased mortality risk from Parkinson’s disease, but not Alzheimer’s disease [29]. Similarly, a meta-analysis of sugar-sweetened beverage consumption in Asian populations found an increased risk of T2DM in individuals before and after adjusting for body mass index [30].

## 4. Ectopic Calcification and Bone Disorders in Patients with PD

Calcium phosphate that is abnormally deposited in soft tissue, such as the mitochondrial calcium phosphate precipitates formed in PD, is a condition associated with dysregulated phosphate metabolism known as ectopic calcification. These calcifications and other bone mineral disorders in patients with PD provide supporting evidence of dysregulated phosphate metabolism and effects from phosphate toxicity. Importantly, dopaminergic neurons in PD are not the only structures in the midbrain susceptible to calcification potentially related to phosphate toxicity. Mineralization of the deep gray matter occurs with age throughout the substantia nigra, globus pallidus, putamen, caudate nucleus, red nucleus, and thalamus [31]. Additionally, calcifications throughout the basal ganglia are more prevalent and severe in PD compared to Alzheimer’s disease or controls [32].

**Figure 2:**
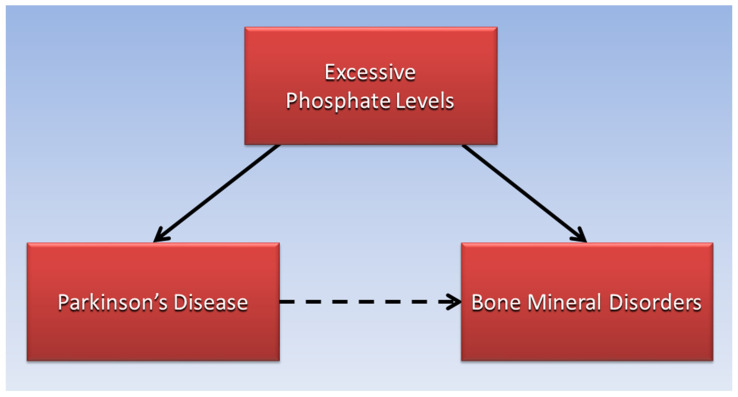
Excessive phosphate levels are proposed to mediate the association of Parkinson’s disease with bone mineral disorders.

Within the skeletal system, “excessive dietary phosphorus has been associated with adverse effects on bone and mineral metabolism” [33]. As serum phosphorus levels rise, parathyroid hormone released from the parathyroid glands and fibroblast growth factor 23 (FGF-23) from bone osteocytes lower serum phosphate by increasing phosphaturia in the kidneys. In the process, calcium is resorbed from bone to maintain serum calcium levels. This mechanism implies that excessive dietary phosphate is also a potential mediating factor in the association of PD with bone mineral disorders, as shown in Figure 2.

For example, a systematic review and meta-analysis of 23 studies concluded that patients with PD have lower bone mineral density (BMD) and higher associated risk for osteoporosis, osteopenia, and fractures than controls [34]. A more recent retrospective, cross-sectional study found that 34 participants aged 60–85 years with PD had lower BMD of the femoral neck and total hip compared with 31 healthy controls of similar age [35]. Another recent study found lower BMD in the femoral neck and lumbar spine as well as lower serum vitamin D levels in 182 patients with PD compared to 185 healthy controls [36], and similar findings of low lumbar and femoral BMD as well as low serum vitamin D were found in a recent study of 124 patients with PD compared to 116 controls [37].

Cataract, an age-related disease and the leading cause of blindness, is a form of ectopic calcification in the crystalline lens of the eye, in which deposits of hydroxyapatite, a component of bone tissue, form from calcium and phosphate [38]. Ectopic calcification in cataract is associated with ROS and with calcification in chronic kidney disease, diabetes, and vascular disease. Of relevance, a retrospective cohort study in Taiwan found that a new diagnosis of cataracts in 26,031 people was associated with a 26% increased risk of PD compared to 25,937 matched individuals without cataracts [39].

Additionally, chronic kidney disease is associated with an increased risk for PD, and chronic kidney disease patients, similar to patients with PD, often suffer dementia and cognitive dysfunction [40]. Of relevance, dysregulated phosphate in chronic kidney disease bone mineral disorder (CKD-BMD) plays a role in vascular calcification as excessive phosphate enters into vascular smooth muscle cells, producing osteochondrocytic transdifferentiation, or vascular ossification [41]. Importantly, as in diabetic vascular calcification related to dysregulated phosphate [42], PD is also associated with a significant increased risk of peripheral vascular disease [43], inferring additional evidence of ectopic calcification from phosphate toxicity in the etiology of PD.

## 5. Vitamin D, Serum Pi, and PD

Vitamin D in the form of 25-hydroxyvitamin D3 is metabolized by 1-alpha hydroxylase in the kidneys to the bioactive form of 1,25-dihydroxyvitamin D3 (calcitriol), which maintains normal serum phosphorus levels by stimulating phosphorus absorption in the intestines [44]. When serum Pi levels rise, FGF-23 decreases vitamin D levels to reduce Pi intestinal absorption [45]. As mentioned previously in Section 4, recent epidemiological studies suggest that patients with PD have lower vitamin D levels than healthy controls [46], inferring downregulation of serum phosphate levels in PD.

By contrast, a recent study found that serum phosphorus levels in 139 patients with PD were significantly lower than in 100 healthy controls, and that “decreased levels of phosphorus resulted in an elevated risk of PD” [47]. However, abnormally low serum phosphorus levels (hypophosphatemia) most commonly occur in hospitalized patients due to a cellular shift and redistribution of inorganic phosphate into intracellular compartments of body tissue [48]. “Serum measurements of extracellular phosphate therefore reveal only a tiny fraction of total body phosphate and might not always reflect the amount of phosphate uptake and its distribution” [49].

Considering that low vitamin D in PD is an endocrine response triggered by high Pi intake, supplementing PD patients with additional vitamin D does not address high Pi intake as a potential contributing cause of PD, which could explain why vitamin D supplementation lacks consistent effectiveness in treating PD. For example, a recent randomized, controlled pilot study found no significant benefit from high-dose vitamin D in improving balance in PD patients [50]. Additionally, a retrospective analysis of data from the National Institutes of Health Exploratory Trials in Parkinson’s Disease found “no difference in early disease progression” observed in PD patients taking vitamin D supplementation [51].

## 6. Sarcopenia, Phosphate Toxicity, and PD

Sarcopenia, low muscle mass, and muscle function increase disability, frailty, morbidity, and mortality and lowers quality of life in the elderly population [52]. Core muscle loss was associated with reduced brain gray matter volume in patients with PD [53]. A recent systematic review and meta-analysis found that “the pooled prevalence of sarcopenia was 29% in PD, which was higher than the healthy older control group” [54]. The researchers suggested that sarcopenia and PD may share a common pathway of neuroinflammation. Coincidentally, a higher prevalence of sarcopenia was found in diabetic compared to non-diabetic individuals in a meta-analysis of an Asian population [55], yet again implying common pathways shared with PD.

Of relevance, increasing dietary Pi fed to lab animals in a model of chronic kidney disease showed a dose-dependent increase in levels of tumor necrosis factor-alpha, a biomarker of systemic inflammation, as well as decreased animal body weight, a biomarker of malnutrition, and increased vascular calcification with reduced lifespan [56]. These findings are consistent with sarcopenia as well as inflammation in PD [57], providing additional support for phosphate toxicity in PD etiology. Moreover, concentrated levels of Pi added to cultured muscle cells directly produced cell autophagy [58], and future studies should investigate muscle cell autophagy associated with phosphate-induced mitochondrial damage, as previously discussed in Section 2.

## 7. Cancer, Phosphate Toxicity, and PD

Tumorigenesis is associated with phosphate toxicity [59], briefly summarized here. As excessive amounts of dysregulated Pi are sequestered into precancerous cells through overexpressed sodium phosphate cotransporters, cell signaling pathways stimulate tumor growth. For example, the phosphoinositide 3 kinase (PI3K) pathway phosphorylates Akt (protein kinase B), leading to activation of mTOR (mammalian target of rapamycin), which upregulates protein synthesis in tumor growth. Phosphorus is a rate-limiting factor in biological growth, and reducing phosphorus transport into a tumor by half is predicted to reduce a tumor’s size by 75% [24].

Positive associations have been found between PD and cancers of the breast, brain, and melanoma, and cancer incidence may occur either before or after PD incidence, which is consistent with a common causative pathway in both of these “pathologically convergent diseases” [60]. Nevertheless, “the lower risks of lung, bladder, and colorectal cancer, all smoking-related cancers, in PD patients are generally undisputed”. Feasibly, lower risks of smoking-related cancers could be explained by the fact that “smokers reported reduced compliance with the DRI [dietary reference intake] for iron, phosphorus, vitamin C, riboflavin, and folate compared to nonsmokers” [61]. With a DRI of 700 mg phosphorus per day for U.S. adults, “the average daily phosphorus intake from foods is 1189 mg for women and 1596 mg for men” [62], implying that smokers’ phosphorus intake is approximately twice as low as the average intake. Moreover, lower phosphorus intake could explain “a causally protective effect of current smoking on the risk of PD” [63].

## 8. High Dietary Phosphate and PD

Globally, “the presence of any known causal PD mutation is rare, occurring in less than 2% of the PD population”, inferring that environmental factors such as diet are likely to play a significant role in PD etiology [64]. Relevant to the potential contribution of phosphate toxicity to PD pathophysiology, “high phosphorus intake is associated with increased mortality in a healthy US population” [65]. Importantly, “dairy products, fish, and other types of meat are a major source of phosphate in the human diet”, and “preservatives and additive salts commonly used in processed foods contain large amounts of phosphate” [66].

A prospective study of the EPIC-Greece cohort found that “incident PD exhibited strong positive association with consumption of milk, but not cheese or yoghurt”, and an “inverse association was found between polyunsaturated fat intake and incident PD” [67]. Milk consumption, but not fermented milk, was also weakly associated with increased risk of PD in a recent Swedish study [68]. Associations between various food items and PD could be mediated by an inverse relationship between the phosphorus and fat content in foods—phosphorus in food is naturally found in combination with protein [69], not with fat. Full-fat dairy products provide more calories and have lower phosphorus caloric densities (lower phosphorus levels per kcal), which helps meet caloric dietary needs with overall less phosphate, compared to low-fat or non-fat dairy products that provide fewer calories and have higher phosphorus caloric densities, which may drive up overall phosphorus intake to meet calorie requirements. Accordingly, a Harvard analysis of data from the Nurses’ Health Study and the Health Professionals’ Follow-Up Study found that three or more servings of low-fat dairy was associated with a higher risk of PD diagnosis, but no association was found with consumption of full-fat dairy [70].

Low phosphorus in a high-fat ketogenic diet (KD) could also explain neuroprotection of the KD, although “the literature does not yet support a neuroprotective effect of the KD in PD” [71]. Nevertheless, current data suggest benefits in non-motor symptoms of PD patients using a ketogenic diet [72]. Additionally, whole-food plant-based diets such as the Mediterranean diet, with reduced Pi intake from animal food products and highly processed foods, have been associated with delayed onset of PD [73]. A recent analysis of dietary patterns in the Rotterdam Study reported reduced associated risks of PD in the Netherlands general population that “corroborate previous findings of a possible protective effect of the Mediterranean diet” [74]. This type of plant-based diet often contains an abundance of whole fruit, which has low phosphorus caloric density. Table 1 shows the phosphorus caloric density of various food items based on data from the U.S. Department of Agriculture (USDA) National Nutrient Database for Standard Reference, Legacy 2018 [75]. Note that whole fruit, although high in sugar, is also high in fiber with a low glycemic index [76], and whole fruit is associated with decreased risk of diabetes [77,78]. Future studies should investigate potential PD benefits specifically associated with lower phosphate intake in whole-food plant-based diets.

Restriction of dietary phosphate, 800–1000 mg/day, is currently used in managing phosphate serum levels in patients with chronic kidney disease [79], and similar strategies should be investigated to prevent and delay progression of PD. Dietary counseling from renal dietitians and other trained healthcare providers “can lead to better control of phosphorus intake” [69]. Importantly, in vivo regression of ectopic calcification is associated with the phosphoprotein osteopontin (OPN) [80], and vascular calcification reversal in rats fed low-phosphate diets was suggested to be linked to OPN [81]. Phosphorylation regulates the inhibitory effect of OPN on vascular calcification [82], providing a potential compensatory response to calcification associated with elevated phosphate levels [83]. This evidence implies that some degree of regression of mitochondrial calcification in PD may be possible by placing patients with PD on restricted-phosphate diets.

## 9. Conclusions

The global burden of PD has more than doubled over the past generation and is predicted to increase substantially. With no known cure, novel approaches are needed to investigate the cause and prevention of PD. Excessive calcium phosphate precipitates are proposed to reduce Complex I function of the mitochondrial electron transport chain in the substantia nigra in PD, and phosphate is linked to opening of the mitochondrial permeability transition pore, which is associated with increased ROS, inflammation, DNA damage, and cell death. PD is also associated with α-synuclein and Lewy body dementia, a secondary tauopathy related to hyperphosphorylation of tau protein. Feasibly, inorganic phosphate may be a rate-limiting factor in tau hyperphosphorylation, and this should be further investigated. Additionally, PD is associated with diabetes prevalence, and tauopathy and mitochondrial dysfunction are pathophysiological mechanisms potentially shared between PD and diabetes, reducing biosynthesis and secretion of dopamine and insulin, respectively. Excessive phosphate from dysregulated phosphate metabolism is also associated with ectopic calcification, bone mineral disorders, and low levels of serum vitamin D in patients with PD. Sarcopenia and cancer in PD patients are also associated with phosphate toxicity. Finally, restricted dietary phosphate intake, used to treat chronic kidney disease, may be effective in treating and preventing PD by reducing phosphate toxicity. Furthermore, osteopontin associated with a low-phosphorus diet may potentially regress calcifications in PD patients. However, no clinical trials have tested a reduced-phosphate diet to treat and prevent PD. More studies are needed to investigate the potential mediating role of phosphate toxicity in the etiology of PD.

## Figures and Tables

**Table 1:** Phosphorus caloric density of selected food items.

Food Item	Phosphorus mg/kcal	Food Item	Phosphorus mg/kcal
Pineapple	0.16	Potato, white	0.90
Pear	0.21	Corn	1.03
Apple	0.21	Wheat flour, wholegrain	1.05
Date, Medjool	0.22	Brazil nut	1.10
Macadamia nut	0.26	Beef, lean	1.54
Coconut	0.32	Fish, Tilapia	1.77
Avocado	0.33	Chicken breast, skinless	1.78
Rice, brown	0.73	Cow milk, non-fat	2.89

Based on data from USDA National Nutrient Database for Standard Reference, Legacy (2018).

## Data Availability

Not applicable.
